# Striatal (a-)symmetry reveals sex-specific autonomic vulnerabilities in early Parkinson’s disease

**DOI:** 10.1038/s44294-025-00121-8

**Published:** 2026-01-06

**Authors:** Anthony Nuber-Champier, Julie Anne Péron

**Affiliations:** 1 Clinical and Experimental Neuropsychology Laboratory, Department of Psychology, University of Geneva, Geneva, Switzerland; 2 Swiss Centre for Affective Sciences, University of Geneva, Geneva, Switzerland; 3 Department of Neurology, University Hospitals of Geneva, Geneva, Switzerland

**Keywords:** Diseases, Health care

## Abstract

Autonomic symptoms are common in Parkinson’s disease and vary according to patterns of dopaminergic neurodegeneration. The impact of sex and striatal denervation asymmetry are interesting avenues for observing distinct patient phenotypes from the early stages of the disease. This exploratory study investigates how striatal denervation asymmetry and sex influence autonomic dysfunction profiles in early-stage, treatment-naive Parkinson’s disease. Using data from the Parkinson’s Progression Markers Initiative (*n* = 759), we applied generalized linear mixed models to assess SCOPA-AUT scores across subtypes of striatal denervation (left-predominant, right-predominant, symmetric), modeling the interaction with sex. Patients with symmetric striatal denervation exhibited significantly greater overall autonomic dysfunction, particularly in sexual domains (*p* = 0.045). Sex-specific effects emerged: women showed more pronounced thermoregulatory symptoms (*p* = 0.001), whereas men exhibited more severe urinary and sexual dysfunction, especially in the symmetric group (*p* < 0.0001). These findings suggest the potential of integrating striatal denervation asymmetry and sex into Parkinson’s disease subtype characterization, with implications for personalized symptom management.

## Introduction

Parkinson’s disease (PD) is a complex neurodegenerative disorder affecting millions worldwide. It is characterized by hallmark motor symptoms and a broad range of non-motor manifestations, including cognitive decline and autonomic dysfunction, which significantly impact quality of life^1,2^. Motor symptoms often begin asymmetrically, reflecting underlying dopaminergic denervation in the striatum^3^. Despite extensive research, the links between the lateralization of striatal neurodegeneration and the heterogeneity of PD symptoms, both motor and non-motor, remain unclear. In recent years, growing attention has been paid to the asymmetry of neurodegeneration as a potential factor shaping disease expression and progression^4–6^. The Synuclein Origin and Connectome (SOC) model, proposed by Borghammer^4^, provides an interesting theoretical framework that distinguishes between two main PD phenotypes: a “brain-first” subtype, characterized by initially asymmetric cerebral involvement, and a “body-first” subtype, marked by more symmetric pathology and prominent autonomic symptoms. However, the role of asymmetry within this model remains debated, and current evidence based solely on dopaminergic imaging does not yet allow clear subtype differentiation^7–9^. Nevertheless, asymmetry remains a relevant dimension for understanding interindividual variability in PD presentation.

Autonomic symptoms are common in PD and can appear early in the disease course^10,11^. They encompass cardiovascular, gastrointestinal, urinary, and thermoregulatory disturbances, and contribute substantially to morbidity and reduced quality of life^12,13^. Understanding how these symptoms vary according to underlying dopaminergic patterns could help clarify mechanisms of dysautonomia in PD. Preliminary work, for example, suggests that left-predominant striatal denervation may be associated with more pronounced gastrointestinal symptoms compared to right-predominant or symmetric denervation^14^ although these findings require replication in larger and more homogeneous samples.

Another factor that may influence PD expression is sex. Increasing evidence indicates that men and women differ in disease risk, progression, and symptom profiles^15,16^. In particular, women often report a greater burden of non-motor and autonomic symptoms, such as gastrointestinal or thermoregulatory disturbances^17,18^. Hormonal differences, especially estrogen modulation, may partly account for these effects, as the decline in estrogen during menopause has been linked to the exacerbation of both motor and non-motor features^19,20^. While only a few studies have investigated how sex may interact with striatal dopaminergic asymmetry to influence autonomic dysfunction in PD, emerging evidence indicates that sex itself affects both the extent and the pattern of dopaminergic denervation, with women typically exhibiting a less pronounced early nigrostriatal loss than men^21,22^. This sex-related discrepancy in dopaminergic degeneration offers a plausible mechanistic basis for considering sex as a modifier of autonomic symptoms in the context of striatal asymmetry.

Taken together, these observations highlight the need to explore how striatal dopaminergic asymmetry and sex jointly shape autonomic symptom profiles in PD.

In this context, and although asymmetry based solely on dopaminergic innervation is still being evaluated and is not consistently supported by the literature, this exploratory study examines how striatal denervation asymmetry and sex shape autonomic dysfunction in early-stage PD, using data from the treatment-naive cohort of newly diagnosed patients in the Parkinson’s Progression Markers Initiative (PPMI).

It is hypothesized that sex and dopaminergic asymmetry jointly influence the presentation of autonomic symptoms in individuals with PD. Specifically, autonomic symptom profiles are expected to differ significantly based on the type of dopaminergic asymmetry (left-sided, right-sided, or symmetric). For example, according to Murtomäki, et al.^12^, left- predominant striatal denervation would be associated with a higher prevalence of gastrointestinal dysautonomia. Sex is also hypothesized to play a critical role, influencing both the type and severity of autonomic symptoms, with women more likely to report gastrointestinal and thermoregulation dysautonomia than men^14,15^. Furthermore, the combination of dopaminergic asymmetry and sex may reveal intergroup differences in the burden of dysautonomia symptoms.

## Results

### Sociodemographic and clinical data

Table 1 showed the sociodemographic and clinical differences according to both the different striatal denervation groups and the sex. We presented these data for a total of 759 patients.

**Table 1:** Sociodemographic and clinical features as a function of striatal (a)symmetry and sex

	Right-predominant striatal denervation (*n* = 133)	Left-predominant striatal denervation (*n* = 192)	Symmetric striatal denervation (*n* = 434)	*p* value
Mean age in years ( ± *SD*)	60.95 ( ± 9.52)	60.94 ( ± 9.54)	64.77 ( ± 8.96)	<0.0001
Mean age in years ( ± *SD*) **women**	61.16 ( ± 9.67)	61.27 ( ± 8.21)	64.42 ( ± 9.64)	0.007
Mean age in years ( ± *SD*) **men**	60.85 ( ± 9.50)	60.78 ( ± 10.17)	64.96 ( ± 8.57)	<0.0001
Men/Women	88/45	128/64	281/153	0.88
Mean years of education ( ± SD)	16.02 ( ± 2.95)	16.17 ( ± 2.83)	16.02 ( ± 3.28)	0.90
Mean years of education ( ± SD) **women**	16.07 ( ± 3.16)	15.78 ( ± 2.53)	15.93 ( ± 3.54)	0.90
Mean years of education ( ± SD) **men**	16.00 ( ± 2.85)	16.37 ( ± 2.96)	16.07 ( ± 3.13)	0.66
Mean time (months) between diagnosis and baseline measurements ( ± *SD*)	8.19 ( ± 7.47)	9.02 ( ± 7.90)	8.37 ( ± 7.41)	0.36
Mean time (months) between diagnosis and baseline measurements ( ± *SD*) **women**	9.12 ( ± 8.56)	8.78 ( ± 8.01)	8.62 ( ± 8.02)	0.94
Mean time (months) between diagnosis and baseline measurements ( ± *SD*) **men**	7.72 ( ± 6.86)	9.14 ( ± 7.87)	8.23 ( ± 7.07)	0.28
Mean putamen denervation (SBR): right ( ± SD)	0.65 ( ± 0.21)	1.20 ( ± 0.40)	0.83 ( ± 0.31)	<0.0001
Mean putamen denervation (SBR): right ( ± SD) **women**	0.68 ( ± 0.25)	1.24 ( ± 0.46)	0.85 ( ± 0.31)	<0.0001
Mean putamen denervation (SBR): right ( ± SD) **men**	0.63 ( ± 0.19)	1.18 ( ± 0.38)	0.83 ( ± 0.31)	<0.0001
Mean putamen denervation (SBR): left ( ± SD)	1.19 ( ± 0.39)	0.65 ( ± 0.23)	0.83 ( ± 0.31)	<0.0001
Mean putamen denervation (SBR): left ( ± SD) **women**	1.27 ( ± 0.42)	0.67 ( ± 0.26)	0.87 ( ± 0.32)	<0.0001
Mean putamen denervation (SBR): left ( ± SD) **men**	1.16 ( ± 0.37)	0.64 ( ± 0.21)	0.81 ( ± 0.31)	<0.0001
Handedness				
Right *n (%)*	114 (85.71%)	173 (90.10%)	383 (88.24%)	0.56
Left *n (%)*	16 (12.03%)	13 (6.77%)	41 (9.44%)	
Ambidextrous *n (%)*	3 (2.25%)	6 (3.12%)	10 (2.30%)	
Handedness **women**				
Right *n (%)*	43 (95.55%)	60 (93.75)	140 (91.50%)	0.73
Left *n (%)*	2 (4.44%)	3 (4.68%)	12 (7.84%)	
Ambidextrous *n (%)*	0	1 (1.56%)	1 (0.65%)	
Handedness **men**				
Right *n (%)*	71 (80.68%)	113 (88.28%)	243 (86.47%)	0.43
Left *n (%)*	14 (15.90%)	10 (7.81%)	29 (10.32%)	
Ambidextrous *n (%)*	3 (3.40%)	5 (3.90%)	9 (3.20%)	
MDS-UPDRS 3 total score (Mean ± *SD*)	34.80 ( ± 14.09)	31.87 ( ± 14.33)	35.68 ( ± 14.96)	0.004
MDS-UPDRS 3 total score (Mean ± *SD*) **women**	35.38 ( ± 14.11)	32.75 ( ± 16.16)	36.44 ( ± 15.14)	0.10
MDS-UPDRS 3 total score (Mean ± *SD*) **men**	34.51 ( ± 14.14)	31.44 ( ± 13.37)	35.27 ( ± 14.87)	0.046
Right-lateralised item in the MDS-UPDRS 3	6.86 ( ± 5.78)	10.34 ( ± 5.01)	7.54 ( ± 4.96)	<0.001
Left-lateralised item in the MDS-UPDRS 3	9.71 ( ± 5.65)	5.60 ( ± 5.42)	7.63 ( ± 5.78)	<0.001
Hoehn and Yahr				
• stage I *n (%)*	46 (34.58%)	91 (47.39%)	130 (29.95%)	0.001
• stage 2 *n (%)*	87 (65.41%)	100 (52.08%)	302 (69.58%)	
• stage 3 *n (%)*	0	1 (0.52%)	2 (0.46%)	
Hoehn and Yahr **women**				
• stage I *n (%)*	18 (40%)	26 (40.62%)	45 (29.41%)	0.15
• stage 2 *n (%)*	27 (60%)	37 (57.81%)	108 (70.58%)	
• stage 3 *n (%)*	0	1 (1.56%)	0	
Hoehn and Yahr **men**				
• stage I *n (%)*	28 (31.81%)	65 (50.78%)	85 (30.24%)	0.001
• stage 2 *n (%)*	60 (68.18%)	63 (49.21%)	194 (69.03%)	
• stage 3 *n (%)*	0	0	2 (0.71%)	

This table shows the sociodemographic and clinical variables of patients according to their allocation groups (based on striatal denervation asymmetry) and sex. MD-UPDRS 3: Movement Disorder Society-Sponsored Revision of the Unified Parkinson’s Disease Rating Scale. The overall differences in proportions between the different conditions (manuality: left-handed, right-handed, ambidextrous; Hoehn and Yahr stage: 1, 2, 3) across the different asymmetry groups (left-predominant striatal denervation, right -predominant striatal denervation, symmetric) — with the same analyses repeated for each sex — were evaluated using the Chi-square test of independence. A single *p*-value is reported, reflecting the differences in proportions across all conditions simultaneously.

Clinical and sociodemographic differences according to the subgroups of interest revealed significant differences in terms of age, handedness and for the MDS-UPDRS 3 total score. Notably, the symmetric group was significantly older and exhibited higher UPDRS scores. Since both age and disease severity are known to influence autonomic dysfunction, these variables were included as covariates in the subsequent GLMM models, the results of which are presented below.

### Dysautonomia symptoms according to striatal (a-)symmetry and sex (*n* = 759)

GLMMs were adjusted for age, laterality, time between diagnosis and the start of the study, education, Hoehn and Yahr stages, and the total MDS-UPDRS III score. The significant results reported survived FDR correction.

### Main effects

#### Left vs Right vs Sym

The group with symmetric striatal denervation had significantly higher total dysautonomia score and higher sexual dysautonomia compared to patients with left-predominant striatal denervation (total score: *p* = 0.045; *t* = 2.01; sexual score: *p* = 0.001; *t* = 3.31) (see Tables 2 and 3).

**Table 2:** Autonomic symptoms among striatal denervation and sex groups

*Group*	*SCOPA-AUT mean (± SD)*	*SCOPA-GI mean (± SD)*	*SCOPA-UR mean (± SD)*	*SCOPA-CAR mean (± SD)*	*SCOPA-THER mean (± SD)*	*SCOPA-PUP mean (± SD)*	*SCOPA-SEX mean (± SD)*
*Right – whole group*	9.76 ± 7.24	2.41 ± 2.35	4.32 ± 3.46	0.44 ± 1.00	1.17 ± 1.40	0.41 ± 0.67	1.02 ± 1.51
• *Right – women*	9.56 ± 6.44	2.51 ± 2.36	4.07 ± 3.16	0.33 ± 0.64	1.33 ± 1.62	0.42 ± 0.81	0.89 ± 1.43
• *Right – men*	9.86 ± 7.64	2.35 ± 2.36	4.44 ± 3.62	0.50 ± 1.14	1.09 ± 1.28	0.40 ± 0.59	1.08 ± 1.55
*Left – whole group*	9.11 ± 6.71	1.90 ± 2.01	4.13 ± 2.88	0.46 ± 0.94	1.21 ± 1.54	0.39 ± 0.67	1.02 ± 1.53
• *Left – women*	9.36 ± 7.01	1.91 ± 2.29	4.05 ± 2.98	0.45 ± 0.92	1.56 ± 1.99	0.47 ± 0.79	0.92 ± 1.28
• *Left – men*	8.99 ± 6.58	1.89 ± 1.86	4.17 ± 2.83	0.47 ± 0.95	1.04 ± 1.23	0.35 ± 0.61	1.07 ± 1.64
*Symmetric – whole group*	10.51 ± 6.51	2.53 ± 2.25	4.71 ± 3.14	0.48 ± 0.76	1.13 ± 1.37	0.40 ± 0.67	1.26 ± 1.69
• *Symmetric – women*	9.62 ± 5.67	2.52 ± 2.25	4.05 ± 2.57	0.41 ± 0.67	1.41 ± 1.48	0.41 ± 0.62	0.83 ± 1.40
• *Symmetric – men*	11.00 ± 6.89	2.54 ± 2.25	5.07 ± 3.35	0.52 ± 0.81	0.99 ± 1.29	0.39 ± 0.69	1.49 ± 1.78

The scores obtained by the *n* = 759 patients on the SCOPA-AUT scale and the sub-dimensions are shown in the table. The terms Left, Right, and Symmetric (Sym) refer to the (a)symmetry of striatal denervation, which is predominantly left, right or symmetric. GLMMs were adjusted for age, laterality, and the total MDS-UPDRS III score. SCOPA-AUT: total dysautonomia score; SCOPA-GI: gastrointestinal dysautonomia score; SCOPA-UR: urinary dysautonomia score; SCOPA-CAR: cardiovascular dysautonomia score; SCOPA-THER: thermoregulatory dysautonomia score; SCOPA-PUP: pupillomotor dysautonomia score; SCOPA-SEX: sexual dysautonomia score.

**Table 3:** Group comparisons of dysautonomia scores according to striatal denervation pattern, sex, and their interaction

*Group contrast*	*SCOPA-AUT*	*SCOPA-GI*	*SCOPA-UR*	*SCOPA-CAR*	*SCOPA-THER*	*SCOPA-PUP*	*SCOPA-SEX*
*Right vs Symmetric*	*p* = 0.45; β = 0.48	*p* = 0.99; β = 0.07	*p* = 0.29; β = −033	*p* = 0.90; β = 0.00	*p* = 0.77; β = 0.04	*p* = 0.58; β = 0.04	*p* = 0.038; β = −017
*Left vs Symmetric*	*p* = **0.045**; β = 1.14	*p* = 0.95; β = −056	*p* = 0.10; β = −045	*p* = 0.82; β = −001	*p* = 0.74; β = 0.04	*p* = 0.59; β = 0.04	*p* = **0.001**; β = −024
*Left vs Right*	*p* = 0.37; β = 0.66	*p* = 0.96; β = 0.63	*p* = 0.74; β = 0.11	*p* = 0.77; β = 0.02	*p* = 0.99; β = 0.00	*p* = 0.94; β = 0.00	*p* = 0.46; β = 0.07
*Men vs Women*	*p* = 0.94; β = 0.03	*p* = 0.98; β = 0.13	*p* = 0.19; β = 0.33	*p* = 0.27; β = 0.06	*p* = **0.001**; β = -.37	*p* = 0.16; β = −010	*p* < **0.0001**; β = 0.51
*Sex × Right*	*p* = 0.74; β = 0.37	*p* = 0.98; β = 0.38	*p* = 0.84; β = 0.11	*p* = 0.71; β = 0.04	*p* = 0.20; β = −031	*p* = 0.20; β = −020	*p* = **0.013**; β = 0.36
*Sex × Left*	*p* = 0.62; β = 0.46	*p* = 0.99; β = 0.02	*p* = 0.91; β = 0.05	*p* = 0.82; β = 0.02	*p* = 0.06; β = −036	*p* = 0.17; β = −017	*p* = **.012**; β = 0.31
*Sex × Sym*	*p* = 0.13; β = 0.95	*p* = 0.99; β = 0.01	*p* = **0.006**; β = 0.85	*p* = 0.08; β = 0.12	*p* = **0.001**; β = −044	*p* = 0.41; β = 0.06	*p* < **0.0001**; β = 0.85

The results of group comparisons ((a)symmetry of striatal denervation, sex) of the *n* = 759 patients according to different dimensions of dysautonomia (SCOPA-AUT) are represented by *p*-values and slope β. The terms Left, Right and Symmetric (Sym) refer to the (a)symmetry of striatal denervation, which is predominantly left, right, or symmetric. GLMMs were adjusted for age, laterality, and the total MDS-UPDRS III score. SCOPA-AUT: total dysautonomia score; SCOPA-GI: gastrointestinal dysautonomia score; SCOPA-UR: urinary dysautonomia score; SCOPA-CAR: cardiovascular dysautonomia score; SCOPA-THER: thermoregulatory dysautonomia score; SCOPA-PUP: pupillomotor dysautonomia score; SCOPA-SEX: sexual dysautonomia score. The significant results reported survived FDR correction (in bold).

#### Men vs women

The women’s group had a significantly higher thermoregulatory dysautonomia scores as compared to men (*p* = .001; *t* = 3.22), while men had significantly higher symptoms of sexual dysautonomia than women (*p* < .0001; *t* = 7.36).

### Interaction effects

#### Urinary dysautonomia

**Fig. 1:**
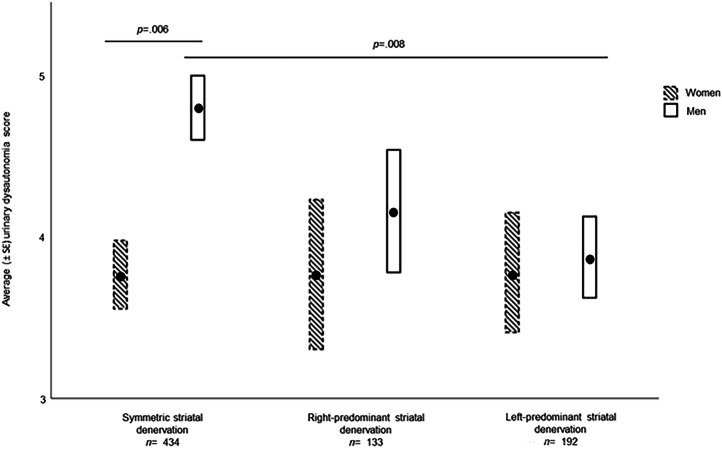
Mean urinary dysautonomia (± SE) as a function of sex and striatal denervation. The differences in urinary dysautonomia symptoms are shown according to striatal denervation asymmetry and sex. GLMMs were adjusted for age, laterality, time between diagnosis and the start of the study, education, Hoehn and Yahr stages, and MDS-UPDRS III score. The significant results reported survived FDR correction. Standard error (SE).

Men with symmetric denervation had significantly more urinary dysautonomia than women with symmetric denervation (*p* = .006; *t* = 2.73) (See Fig. 1).

#### Sexual dysautonomia

**Fig. 2:**
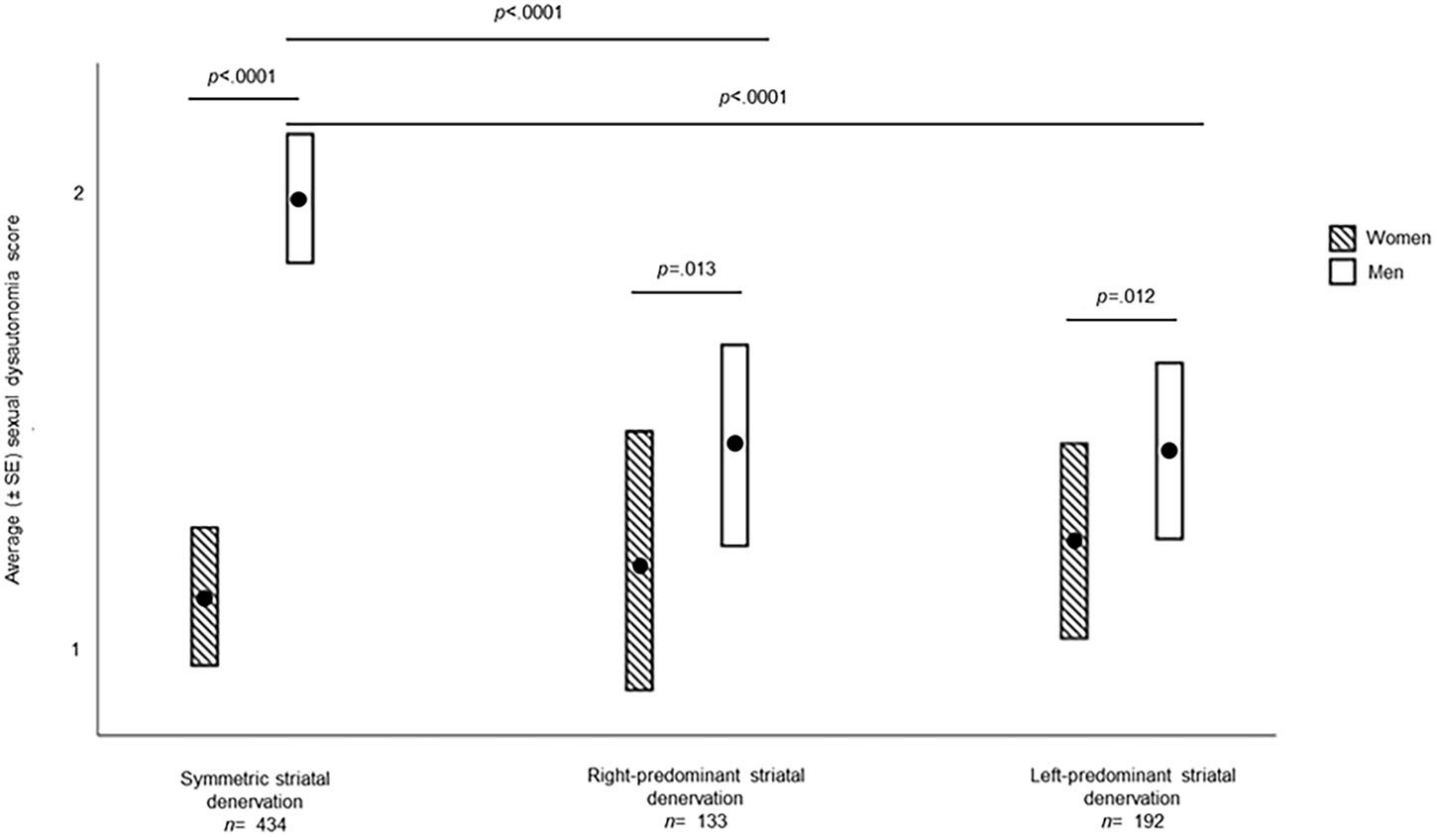
Mean sexual dysautonomia (± SE) as a function of sex and striatal denervation. The differences in sexual dysautonomia symptoms are shown according to striatal denervation asymmetry and sex. GLMMs were adjusted for age, laterality, time between diagnosis and the start of the study, education, Hoehn and Yahr stages, and total MDS-UPDRS III score. The significant results reported survived FDR correction. Standard error (SE).

Men with symmetric striatal denervation (*p* < .0001; *t* = 10.90), right-predominant striatal denervation (*p* = .013; *t* = 2.49) or left-predominant striatal denervation (*p* = .012; *t* = 2.50) had significantly more sexual dysautonomia than their women counterparts (See Fig. 2).

#### Thermoregulatory dysautonomia

**Fig. 3:**
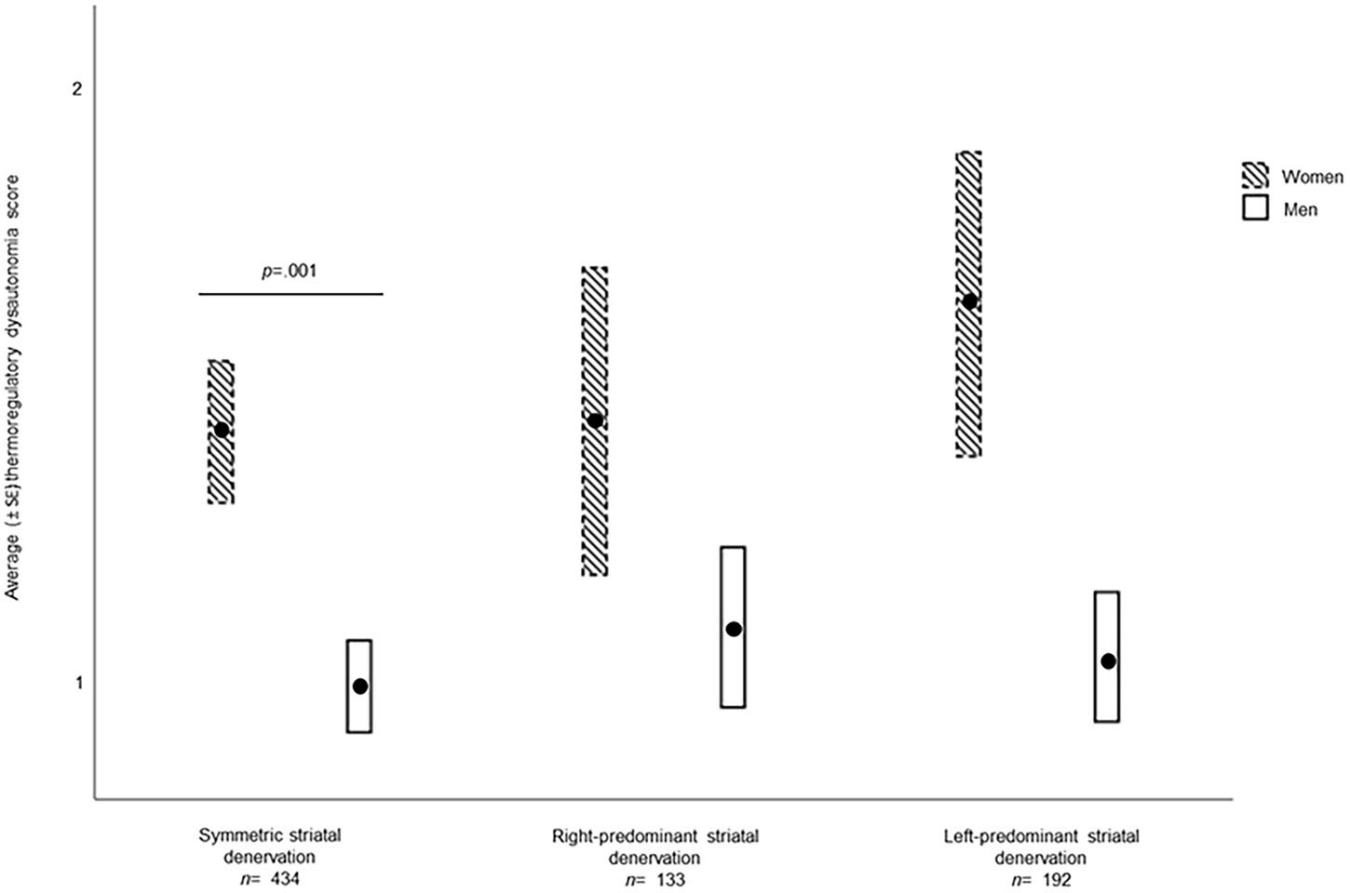
Mean thermoregulatory dysautonomia (± SE) as a function of sex and striatal denervation. The differences in thermoregulatory dysautonomia symptoms are shown according to striatal denervation asymmetry and sex. GLMMs were adjusted for age, laterality, time between diagnosis and the start of the study, education, Hoehn and Yahr stages, and total MDS-UPDRS III score. The significant results reported survived FDR correction. Standard error (SE).

Women with symmetric denervation had significantly more thermoregulatory dysautonomia than men with symmetric denervation (*p* = .001; *t* = 3.21) (See Fig. 3). No other significant results were observed.

## Discussion

In this exploratory study, we investigated how striatal denervation asymmetry and sex interact to influence autonomic dysfunction in early-stage, treatment-naive PD. Our findings partially support the initial hypotheses. First, patients with symmetric striatal denervation exhibited higher overall autonomic dysfunction, compared to those with asymmetric denervation, suggesting a distinct clinical phenotype associated with the patterns of dopaminergic degeneration. Second, sex-related differences emerged across specific autonomic domains: women reported greater thermoregulatory dysfunction, while men experienced more severe sexual and urinary symptoms, particularly in the symmetric denervation group. Contrary to expectations^12^, no significant differences were found in gastrointestinal dysautonomia between asymmetry subtypes or between sex. These findings indicate that both striatal denervation patterns and sex significantly shape the autonomic symptom burden in early PD, though not always in line with previous literature. Below, we discuss our findings in relation to the original hypotheses, their integration into current theoretical models, clinical implications, and study limitations.

Our results confirm the main hypothesis that striatal denervation asymmetry on DAT-SPECT scan is associated with variations in dysautonomia. Specifically, and controlling age and total MDS-UPDRS scores, patients with symmetric striatal denervation exhibit significantly higher overall dysautonomia scores compared to those with left-predominant denervation. This finding is consistent with previous studies indicating that symmetric degeneration is associated with more pronounced non-motor symptoms, including dysautonomia^4,5^. In this sense, we also observed that patients with symmetric striatal denervation have significantly higher sexual dysautonomia scores than those with left-predominant striatal denervation. Interestingly, these findings may be relevant for the investigation of non-motor symptoms in PD within the SOC model.

Contrary to previous studies^12,15^, our results did not reveal significant group differences in gastrointestinal, cardiovascular, or pupillomotor dysautonomia. These domain-specific null findings warrant critical evaluation. Regarding gastrointestinal symptoms, discrepancies with prior work, such as the study by Murtomäki, et al. ^12^, may stem from methodological and clinical differences. Their study used different asymmetry metrics, assessed dysautonomia with other scales, and included patients with longer disease duration and dopaminergic treatment. In contrast, our cohort was drug-naive, with a mean disease duration of 8.5 months, suggesting that gastrointestinal dysautonomia may reflect later disease stages or treatment effects. For cardiovascular and pupillomotor symptoms, neuroanatomical and methodological factors may explain the absence of significant associations with striatal asymmetry or sex. These domains are regulated by brainstem and hypothalamic structures, such as the nucleus tractus solitarius, dorsal motor nucleus of the vagus, and midbrain areas^16^, less directly involved in striatal dopaminergic circuits and not adequately captured by DAT-SPECT imaging. Moreover, the SCOPA-AUT scale may lack sensitivity to detect subtle or early abnormalities in these systems. Objective measures such as orthostatic heart rate variability or pupillometry would offer more precise evaluation. It is also possible that these autonomic domains follow different temporal trajectories: thermoregulatory and urinary symptoms may emerge earlier, while cardiovascular and pupillomotor dysfunction might manifest later or under different patterns. Individual variability and compensatory mechanisms, such as differences in vagal tone, could further obscure clinical detection in early-stage PD. These findings underscore the importance of using multimodal and domain-specific tools to better characterize autonomic dysfunction in relation to disease phenotype, asymmetry, and sex.

The differential impact of sex on autonomic symptoms across asymmetry types underscores the importance of integrating both sex and striatal denervation asymmetry (i.e., left versus right) into the clinical framework of PD. For example, in our study, women with symmetric striatal denervation exhibited significantly higher thermoregulatory dysautonomia compared to their male counterparts. The elevated thermoregulatory dysautonomia among women may also reflect a heightened sensitivity of autonomic systems linked to hormonal and physiological differences. These results are consistent with previous literature suggesting that women are more prone to autonomic disturbances, including thermoregulatory dysautonomia, in PD^17^. Hormonal differences, particularly the effects of estrogen, may underlie this disparity. Estrogen has been implicated in modulating autonomic nervous system activity, and its decline during menopause may exacerbate autonomic symptoms^18^. As observed in PD in other studies, the effects of estrogen may be protective against dementia associated with the disease^19^, and hormone stimulation could slow cognitive decline without preventing the onset of PD^20^. These findings underscore the importance of considering hormonal status in evaluating and managing dysautonomia in women with PD. Women with symmetric striatal degeneration and the most severe thermoregulatory difficulties could be a target population for hormone replacement therapy trials. Hormone therapy could have an impact on thermoregulation through effects on the central nervous system and have peripheral vasodilatory effects^23^.

Conversely, men with symmetric denervation display more urinary and sexual dysautonomia than women, suggesting potential sex-specific autonomic vulnerabilities^24^. This aligns with reports that men with PD may experience more severe urinary dysautonomia than women^15^. In this sense, it would appear that various sex hormones could impact the nigrostriatal pathways, as may be the case with testosterone. Indeed, testosterone treatment in deficient patients could improve non-motor symptoms, particularly sexual and cognitive symptoms^25^. Additionally, the increased prevalence of sexual disorders involved in the profiles of the groups with left and right predominant striatal denervation, supports the hypothesis that asymmetric nigrostriatal degeneration is not merely a motor phenomenon but one with critical autonomic implications. When coupled with sex, this asymmetry appears to stratify patients into further subtypes with distinct autonomic symptom trajectories. That being said, it is important to acknowledge that the SCOPA-AUT scale may have limitations in accurately assessing sexual dysfunction in women. While the scale includes items related to sexual interest and activity, it predominantly reflects male-specific aspects, such as erectile function, and may overlook common female-specific issues like vaginal dryness, pain during intercourse, or difficulties achieving orgasm, loss of libido, and involuntary vaginal contractions. This potential measurement bias could lead to an underestimation of sexual dysautonomia in women and may partly explain the higher sexual dysfunction scores observed in men across all striatal denervation subtypes. Future studies should consider using more comprehensive and sex-sensitive tools to better capture the full spectrum of sexual dysfunction in PD.

The SOC model^8^, provides an excellent foundation for understanding PD heterogeneity^4^, but our findings suggest that sex, striatal denervation patterns may represent additional variables that can refine its application to personalized medicine. Moreover, our results suggest potential avenues for tailoring management strategies. For patients showing symmetric denervation, early intervention focusing on autonomic dysfunction may be particularly beneficial. Hormonal therapies or sex-specific approaches to manage thermoregulatory and urinary symptoms could also prove effective, and future research should consider stratifying participants by sex and asymmetry pattern, while also exploring the potential role of sex hormones, to better understand the mechanisms underlying these variations and to refine patient-specific approaches to care in PD.

Several limitations are associated with this work. First, the cross-sectional design does not allow conclusions to be drawn about the causality of the phenomena or about the generalization of the various possible developments after the early stage. Longitudinal studies are necessary to evaluate how the relationship between asymmetry, sex, and autonomic symptoms evolves over time. Second, the study relies exclusively on the SCOPA-AUT, a self-report questionnaire, which, while widely used, may not capture the full spectrum of dysautonomia. This limitation is particularly relevant when considering the variability of symptoms related to biological hormonal fluctuations or the use of hormonal contraceptives, which may have long-term effects on sexual and thermoregulatory functions, especially in women with PD^26,27^. In addition, the sexual dysfunction subscale of the SCOPA-AUT may be less sensitive to female-specific concerns, potentially contributing to the observed sex differences PD. Future studies could benefit from the integration of objective tests of autonomic function, such as measurements of orthostatic heart rate variability^28^, gastrointestinal manometry^29^, pupillary light reflexes^30^ or urinary flow rate^31^, in order to validate these results, combined with measures specific to each sex. For example, Miller-Patterson, et al. ^24^ have suggested that different urinary mechanisms may affect women and men differently. Women are more likely to suffer from urinary incontinence, while men are more likely to experience incomplete bladder emptying and weak urinary flow. Moreover, the interaction of hormonal status with dysautonomia is not explicitly examined. Given the potential role of estrogen in modulating autonomic symptoms^32,33^, future research should explore this interaction in more depth with scales more suited to the specificities of dysautonomic disorders according to the population, particularly in postmenopausal women^34^. Finally, although age and motor symptoms were included as covariates in the GLMM models, residual confounding cannot be entirely ruled out, particularly given the cross-sectional nature of the analysis, underscoring the need for replication in more diverse populations and with longitudinal designs.

In summary, this study suggests the potential role of striatal denervation asymmetry and sex in defining subjective symptom profiles of dysautonomia in PD. The findings support the need for personalized approaches to PD management that consider striatal denervation patterns and sex-specific symptomatology for non-motor dysfunctions. By refining our understanding of these interactions, future research may pave the way for more targeted and effective therapeutic strategies, ultimately improving patient outcomes.

## Methods

### Participants

#### Participant criteria

The dataset used in this article was obtained from the PPMI database (www.ppmi-info.org/data)^35^. The global PPMI cohort is composed of male, and female patients diagnosed with PD by a neurologist less than two years before the start of the study, over 30 years of age, not taking any medication. PPMI Participants met the following clinical criteria including either asymmetric resting tremor or asymmetric bradykinesia, or at least two of the following motor signs: bradykinesia, rigidity, and resting tremor^35^. For updated information on this study, see www.ppmi-info.org. Baseline data from sporadic patients were collected (no dopaminergic treatment had been introduced yet). Participants with data available for analysis of SCales for Outcomes in PArkinson’s disease - Autonomic Dysfunction (SCOPA-AUT scale)^36^ and dopamine transporter single-photon emission computed tomography (DAT-SPECT) were retained. This stage resulted in the selection of 759 patients with early-stage PD, comprising 262 women and 497 men.

### Striatal asymmetry calculation and categorization of group

#### Method

Analysis of striatal asymmetry was based on PPMI^37^, Kaasinen^38^ preliminary calculations of specific binding ratios (SBR). As a reminder, the methodology for calculating the SBR is defined as the ratio between the specific signal measured in a region of interest (e.g., the putamen or caudate nucleus) and the non-specific signal measured in a reference region (commonly the occipital cortex). It is defined by the following formula: [(target region/reference region)/ reference region] where target denotes the mean count density within the region of interest and reference the mean count density within the reference (non-specific) region. In the present study, we used the following formula: [(striatal region count density/occipital count density)/ occipital count density]. Thus, the SBR reflects the relative excess of specific binding of dopaminergic transporters in the striatum compared to the non-specific background signal in the occipital cortex. As in Fiorenzato, et al. ^39^ study, we calculated cerebral asymmetry using a standardized score based on the difference in putamen SBRs, according to the following formula: [(right-left putamen SBR)/ (right + left putamen SBR)]*100. We applied a cut-off similar to Fiorenzato and Antonini^20^, although the method for quantifying asymmetry could be refined in future studies^10^. Putaminal asymmetry was defined as a standardized ratio >20% or < −20%^10,39^, with values within this range considered symmetric. Therefore, people with asymmetry greater than 20% were determined to have left-predominant striatal denervation, between 20% and -20% as having symmetric striatal denervation, and people with striatal asymmetry of -20% were classified as having predominantly right-predominant striatal denervation.

#### Groups

The groups were therefore composed of those with striatal asymmetric denervation (*n* = 325) or symmetric denervation (*n* = 434), with the asymmetric group further divided into left-predominant striatal denervation (*n* = 192) and right-predominant striatal denervation (*n* = 133).

### Data selection and extraction

We extracted the following sociodemographic and clinical data.

#### Participant information

Age, sex, years of education, handedness, time from diagnosis to the start of the study, Movement Disorder Society-Sponsored Revision of the Unified Parkinson’s Disease Rating Scale (MDS-UPDRS 3)^40^ scores and Hoehn and Yahr stages.

#### Autonomic Symptom

Autonomic symptoms were assessed using the SCOPA-AUT scale^36^ within the PPMI cohort. The SCOPA-AUT score ranges from 0 to 25 and covers several subdomains, including gastrointestinal, urinary, cardiovascular, thermoregulatory, pupillomotor, and sexual symptoms, providing a comprehensive evaluation of dysautonomia in PD.

### Statistical analysis

The normality of data distribution was tested using the Kolmogorov-Smirnov test. The statistical significance threshold was set at *p* < 0.05 with adjustments using the Benjamini-Hochberg false discovery rate (FDR) correction. Analyses were performed using SPSS version 27.0.0.0.a.

#### Socio-demographical and clinical data

Kruskal–Wallis tests were used to compare continuous variables (e.g., age), and Chi-square tests to compare categorical variables (e.g., handedness). These analyses were first performed considering only the type of striatal denervation, and then repeated by taking into account both the asymmetry of striatal denervation and sex (see Table 1).

#### Differences in autonomic symptoms as a function of striatal denervation and sex

A Generalized Linear Mixed Model (GLMM) was used to analyze the autonomic symptom scores measured by the SCOPA-AUT scale, treated as continuous variables. Fixed effects included sex (male, female), type of striatal denervation (left, right, symmetric), and their combinations (sex x dopaminergic asymmetry). A random effect (factors controlled for in the models) for age, handedness, time between diagnosis and start of study, education, MD-UPDRS III total scores and Hoehn and Yahr stages was added to account for inter-individual variability and within-subject correlations. This approach allowed for the evaluation of both fixed and random factors influencing autonomic symptom scores.

Differences between asymmetry groups within the same sex and power analysis are presented in supplementary Text S1 and supplementary Table S1–S7.

### Ethics

The study was conducted in accordance with the Declaration of Helsinki and Good Clinical Practice (GCP) guidelines of the United States Code of Federal Regulations. All participants in the PPMI clinical database were capable of providing informed consent, in line with the International Conference on Harmonization (ICH) and local regulations. The PPMI study is registered at ClinicalTrials.gov (NCT01141023). Prior to initiation, each participating site obtained approval from its local ethics committee (IRB/IEC). Written informed consent for research participation was obtained from all subjects.

## Supplementary information

Supplementary materials

## Data Availability

Data used in the preparation of this article were obtained on [2020–04–01] for cognitive and DAT-SPECT data from the Parkinson’s Progression Markers Initiative (PPMI) database (https://www.ppmi-info.org/access-data-specimens/download-data), RRID:SCR\_006431. For up-to-date information on the study, visit http://www.ppmi-info.org.
