# Supplementary material for: Striatal (a-)symmetry reveals sex-specific autonomic vulnerabilities in early Parkinson’s disease
Source: NPJ Womens Health. 2026 Jan 6;4(1):2. doi: 10.1038/s44294-025-00121-8 (PMC12768963; doi:10.1038/s44294-025-00121-8)
Supplement: Supplementary file 1 — Supplementary materials [file 44294_2025_121_MOESM1_ESM.docx]

**Supplementary materials**

**Text S1. Power analysis**

To achieve the desired statistical power (1 − β) of 80% and a risk of Type I error (α) of 0.05, results indicated that, based on the estimated Cohen’s d of approximately 0.615 from Yoo, et al. ^1^, 42 participants would be needed in each group.

The following parameters were set: α = 0.05 (two-sided), z1−α/2=1.96z, Power = 0.80 → β = 0.20, z1−β=0.842, *d* = 0.615

(1)

$$n=\frac{{2(z1-\frac{\alpha}{2}+z1-\beta)}^{2}}{d^{2}}$$

(2)

$$n=\frac{{2\left( 1.96+0.842 \right)}^{2}}{{0.615}^{2}}$$

(3)

$$n=41.49$$

**Table S1. Differences between asymmetry groups within the same sex (GLMM analysis)**

Total SCOPA-AUT

| Sex | Contrasts | β | Standard Error | t | *p*-value |
| --- | --- | --- | --- | --- | --- |
|  |  |  |  |  |  |
| Women | Sym - Right | -,180 | 1,062 | -,170 | ,865 |
|  | Sym - Left | ,437 | ,930 | ,471 | ,638 |
|  | Left - Right | ,618 | 1,207 | ,512 | ,609 |
| Men | Sym - Right | 1,151 | ,759 | 1,516 | ,130 |
|  | Sym - Left | 1,855 | ,658 | 2,817 | **,005** |
|  | Left - Right | ,704 | ,848 | ,829 | ,407 |

Legend. Results of intra-group comparisons of the n=759 patients according to total score of dysautonomia (SCOPA-AUT).

**Table S2. Differences between asymmetry groups within the same sex (GLMM analysis)**

SCOPA-GI

| Sex | Contrasts | β | Standard Error | t | *p*-value |
| --- | --- | --- | --- | --- | --- |
|  |  |  |  |  |  |
| Women | Sym - Right | -,249 | 19,506 | -,013 | ,990 |
|  | Sym - Left | ,586 | 17,024 | ,034 | ,973 |
|  | Left - Right | ,835 | 22,080 | ,038 | ,970 |
| Men | Sym - Right | ,106 | 13,809 | ,008 | ,994 |
|  | Sym - Left | ,541 | 11,870 | ,046 | ,964 |
|  | Left - Right | ,434 | 15,287 | ,028 | ,977 |

Legend. Results of intra-group comparisons of the n=759 patients according to gastrointestinal score of dysautonomia (SCOPA-AUT).

**Table S3. Differences between asymmetry groups within the same sex (GLMM analysis)**

SCOPA-UR

| Sex | Contrasts | β | Standard Error | t | *p*-value |
| --- | --- | --- | --- | --- | --- |
|  |  |  |  |  |  |
| Women | Sym - Right | -,042 | ,517 | -,081 | ,935 |
|  | Sym - Left | ,048 | ,452 | ,106 | ,916 |
|  | Left - Right | ,090 | ,587 | ,153 | ,878 |
| Men | Sym - Right | ,703 | ,369 | 1,904 | ,057 |
|  | Sym - Left | ,851 | ,320 | 2,662 | **,008** |
|  | Left - Right | ,148 | ,412 | ,359 | ,720 |

Legend. Results of intra-group comparisons of the n=759 patients according to urinary score of dysautonomia (SCOPA-AUT).

**Table S4. Differences between asymmetry groups within the same sex (GLMM analysis)**

SCOPA-CAR

| Sex | Contrasts | β | Standard Error | t | *p*-value |
| --- | --- | --- | --- | --- | --- |
|  |  |  |  |  |  |
| Women | Sym - Right | -,048 | ,121 | -,402 | ,688 |
|  | Sym - Left | -,038 | ,103 | -,369 | ,712 |
|  | Left - Right | ,011 | ,135 | ,079 | ,937 |
| Men | Sym - Right | ,030 | ,086 | ,349 | ,728 |
|  | Sym - Left | ,066 | ,071 | ,919 | ,359 |
|  | Left - Right | ,036 | ,094 | ,380 | ,704 |

Legend. Results of intra-group comparisons of the n=759 patients according to cardiovascular score of dysautonomia (SCOPA-AUT).

**Table S5. Differences between asymmetry groups within the same sex (GLMM analysis)**

SCOPA-THER

| Sex | Contrasts | β | Standard Error | t | *p*-value |
| --- | --- | --- | --- | --- | --- |
|  |  |  |  |  |  |
| Women | Sym - Right | ,023 | ,231 | ,098 | ,922 |
|  | Sym - Left | -,004 | ,199 | -,022 | ,983 |
|  | Left - Right | -,027 | ,261 | -,103 | ,918 |
| Men | Sym - Right | -,104 | ,164 | -,633 | ,527 |
|  | Sym - Left | -,076 | ,141 | -,539 | ,590 |
|  | Left - Right | ,028 | ,184 | ,152 | ,880 |

Legend. Results of intra-group comparisons of the n=759 patients according to thermoregulation score of dysautonomia (SCOPA-AUT).

**Table S6. Differences between asymmetry groups within the same sex (GLMM analysis)**

SCOPA-PUP

| Sex | Contrasts | β | Standard Error | t | *p*-value |
| --- | --- | --- | --- | --- | --- |
|  |  |  |  |  |  |
| Women | Sym - Right | -,188 | ,150 | -1,252 | ,211 |
|  | Sym - Left | -,169 | ,129 | -1,303 | ,193 |
|  | Left - Right | ,019 | ,176 | ,108 | ,914 |
| Men | Sym - Right | ,081 | ,098 | ,825 | ,410 |
|  | Sym - Left | ,077 | ,086 | ,899 | ,369 |
|  | Left - Right | -,003 | ,109 | -,031 | ,976 |

Legend. Results of intra-group comparisons of the n=759 patients according to pupillomotor score of dysautonomia (SCOPA-AUT).

**Table S7. Differences between asymmetry groups within the same sex (GLMM analysis)**

SCOPA-SEX

| Sex | Contrasts | β | Standard Error | t | *p*-value |
| --- | --- | --- | --- | --- | --- |
|  |  |  |  |  |  |
| Women | Sym - Right | -,071 | ,135 | -,523 | ,601 |
|  | Sym - Left | -,027 | ,119 | -,226 | ,822 |
|  | Left - Right | ,044 | ,157 | ,280 | ,780 |
| Men | Sym - Right | ,418 | ,099 | 4,232 | **<.0001** |
|  | Sym - Left | ,517 | ,087 | 5,920 | **<.0001** |
|  | Left - Right | ,099 | ,114 | ,869 | ,385 |

Legend. Results of intra-group comparisons of the n=759 patients according to sexual score of dysautonomia (SCOPA-AUT).

**Reference:**

1 Yoo, H. S. *et al.* Clinical and dopamine depletion patterns in hyposmia-and dysautonomia-dominant Parkinson’s disease. *Journal of Parkinson’s Disease* **11**, 1703-1713 (2021).
